# Une localisation exceptionnelle de la tuberculose vertébrale Mal de Pott sous-occipital

**DOI:** 10.11604/pamj.2013.14.163.2450

**Published:** 2013-04-25

**Authors:** Sana Yahyaoui, Senda Majdoub, Houneida Zaghouani, Hosni Ben Fradj, Dejla Bakir, Elyes Bouajina, Chakib Kraiem

**Affiliations:** 1 Service d'imagerie médicale, Hôpital universitaire Farhat Hached, Sousse 4000, Tunisie; 2 Service de rhumatologie, Hôpital universitaire Farhat Hached, Sousse 4000, Tunisie

**Keywords:** Tuberculose, mal de pott sous occipital, spondylite, tomodensitométrie, imagerie par résonance magnétique, Tuberculosis, sub-occipital Pott disease, spondylitis, CT scan, MRI

## Abstract

Le mal de Pott est la forme la plus commune de la tuberculose osseuse touchant essentiellement le rachis dorso-lombaire. La localisation sous-occipitale reste exceptionnelle. Le diagnostic de cette entité est le plus souvent tardif ce qui expose à des complications graves. Les radiographies standard ne sont parlantes qu’à un stade tardif de la maladie, d'où l'intérêt de l'imagerie moderne notamment la tomodensitométrie (TDM) et l'imagerie par résonance magnétique (IRM) qui permettent un diagnostic précoce. Nous rapportons un nouveau cas de tuberculose sous-occipitale. Le diagnostic était posé sur l'imagerie en coupe et confirmé histologiquement à la biopsie transorale. Sont rappelés les aspects en imagerie de cette localisation particulière du mal de Pott.

## Introduction

Le mal de Pott sous-occipital ou du rachis cervical supérieur est défini par l'atteinte tuberculeuse des deux premières vertèbres cervicales et des articulations occipito-atloidiennes et atloido-axoidiennes [1]. Cette localisation est rare. Son pronostic, conditionné par l'atteinte bulbo-médullaire et les difficultés diagnostiques de cette entité, soulignent l'intérêt du recours à des moyens d'imagerie moderne afin d'orienter le diagnostic et d'instaurer en conséquence une thérapeutique adaptée. Notre travail s'intéresse aux particularités en imagerie de cette forme à la lumière d'une nouvelle observation.

## Patient et observation

**Figure 1:**
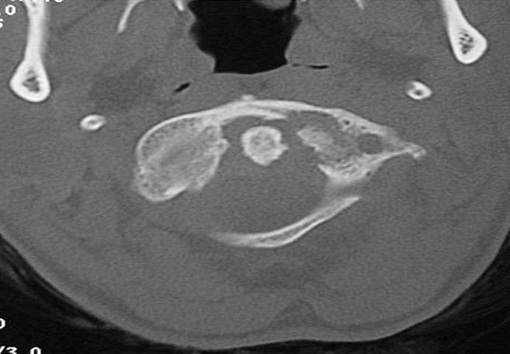
Tomodensitométrie (TDM) de la charnière cervico-occipitale coupe axiale en fenêtre osseuse ostéolyse mal limitée de l'arc antérieur et de la masse latérale gauche de C1.

Mme M.M, âgée de 63ans, d'origine tunisienne, sans antécédents pathologiques notables, consultait pour des cervicalgies. Son histoire de la maladie remontait à un an par l'apparition progressive de rachialgies cervicales mécaniques sans irradiation particulière. L’évolution était marquée par l'accentuation de la douleur et l'apparition récente d'une raideur cervicale avec un retentissement important sur les activités quotidiennes. Cette symptomatologie s'associait à un amaigrissement important de 22Kg et une anorexie. L'examen clinique trouvait une patiente apyrétique et notait une attitude guindée de la tête avec une limitation de tous les mouvements de l'extrémité céphalique. La palpation objectivait la présence d'un ganglion cervical jugulo-carotidien gauche. La biologie était normale notamment l'absence d'un syndrome inflammatoire biologique. La radiographie pulmonaire était normale. Le bilan radiologique standard du rachis cervical de face et de profil n’était pas contributif. La tomodensitométrie (TDM) de la base du crâne objectivait un processus ostéolytique mal limité de l'atlas intéressant son arc antérieur et sa masse latérale gauche associé à une tuméfaction des parties molles de voisinage (Figure 1).

**Figure 2:**
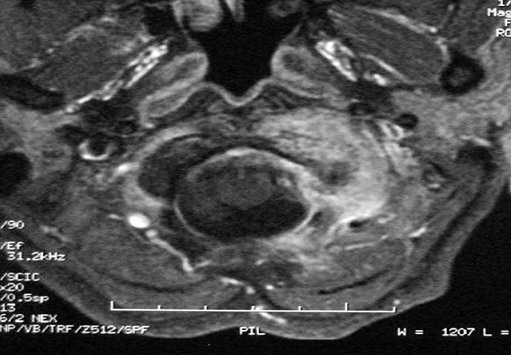
Imagerie par résonance magnétique (IRM) cervicale coupe axiale en séquence pondérée T1+gadolinium prise de contraste intense et hétérogène de l'arc antérieur et de la masse latérale gauche de C1 s’étendant aux parties molles para-vertébrales sans collection.

L'imagerie par résonance magnétique (IRM) confirmait l'analyse scannographique et mettait en évidence une importante infiltration des parties molles pré-vertébrales qui étaient le siège d'une prise de contraste intense et hétérogène sans collection notable (Figure 2). Une biopsie d'un ganglion cervical et une biopsie chirurgicale de l'arc antérieur de l'atlas par voie trans-orale montraient une hyperplasie mixte réactionnelle au niveau du ganglion examiné et une inflammation chronique de type granulomateuse avec nécrose caséeuse évoquant une origine tuberculeuse. Un traitement par quadruple chimiothérapie antituberculeuse était alors instauré pendant quatre mois suivi d'une bithérapie pendant douze mois. Des scanners de contrôle montraient une évolution favorable avec un début de reconstruction osseuse.

## Discussion

La tuberculose ostéo-articulaire reste un problème de santé internationale d′actualité. Son incidence est en recrudescence depuis une quinzaine d′années dans les pays industrialisés du fait de l′augmentation du nombre de patients immunodéprimés (immunosuppresseurs, infection à VIH, vieillissement de la population’), du brassage des populations et de la dégradation des conditions socio-économiques. On assiste, de plus, au développement de souches résistantes [1].

La localisation sous-occipitale de la tuberculose rachidienne est très rare. Elle est estimée à seulement 1% des localisations rachidiennes [2]. Sa pathogénie est plus floue et deux théories sont avancées atteinte osseuse primitive des masses latérales de C1 s′accompagnant d′une destruction du ligament transverse avec extension de l′infection vers l′espace rétropharyngé et les articulations voisines ou atteinte tuberculeuse rétropharyngée primitive suivie d′une extension à C1-C2 par le biais d′anastomoses lymphatiques et veineuses pharyngo-périodontales [1]. Cette forme s′accompagne d′un risque d′atteinte médullaire haute soit par luxation C1-C2 (rupture du ligament transverse) soit par impression basilaire aiguë (écartement des masses latérales de C1 détruites) soit enfin par extension d′un abcès froid épidural antérieur [3, 4]. Le tableau clinique est caractérisé souvent par des cervicalgies d′allure mécaniques au début qui deviennent à caractère inflammatoire au cours de l’évolution de la maladie. Les signes généraux et le syndrome infectieux peuvent être absents [5].

Les radiographies standards de la charnière cervico-occipitale et du rachis cervical peuvent retarder le diagnostic à cause des difficultés d'interprétation [6]. Il s′agit de lésions ostéolytiques allant de la simple érosion à de véritables destructions osseuses qui intéressent en particulier les masses latérales de l′atlas [7]. Dans notre cas, le bilan radiologique standard n’était pas contributif d'où l'intérêt du recours à des moyens d'imagerie en coupe.

La tomodensitométrie permet une meilleure analyse des lésions tuberculeuses suspectées à la radiographie standard. La destruction osseuse est bien analysée en scanner. On en décrit quatre types fragmentation osseuse avec nombreux séquestres (type 1); atteinte lytique pure (type 2); lyse marginale sous-périostée (type 3); et aspect lytique localisé avec sclérose marginale ou « géode centro-corporéale » (type 4). Le type 1 serait le plus fréquent et le plus caractéristique. L′association d′une lésion de type 1 avec un abcès péri-vertébral contenant des fragments osseux serait, pour certains, quasi pathognomonique d′une atteinte tuberculeuse [1]. Elle a également l′avantage de rechercher un abcès ou une masse épidurale, et de guider une ponction ou une biopsie osseuse [5]. L′IRM supplante largement le scanner dans le diagnostic précoce de la tuberculose rachidienne. Il s′agit en effet de l′examen d′imagerie le plus sensible (96%) pour détecter cette pathologie au début elle détecte effectivement précocement l′atteinte du spongieux qui survient avant celle des travées osseuses alors accessibles à la TDM puis aux clichés simples [1]. Dans cette forme, L′IRM permet de mieux visualiser l′atteinte des parties molles surtout les collections pré-vertébrales et leur extension. Ces abcès se manifestent sur les coupes sagittales pondérées en T1 et en T2 sous forme d'un processus de signal hétérogène (en isosignal T1 et en hypersignal T2) [7]. La prise de contraste après injection IV de Gadolinium permet de délimiter les zones hyperhémiées des zones abcédées qui ne se rehaussent pas [1]. Certains abcès tuberculeux peuvent être en hyposignal T2, compte tenu de la présence de fibrine et de caséum [8]. Cependant, à l′opposé du scanner, L′IRM ne permet pas de visualiser les éventuelles calcifications au sein des abcès. Ces calcifications sont importantes car leur présence est inhabituelle dans les abcès non-tuberculeux [2]. Chez notre patiente, l'aspect en IRM était moins évocateur de l'origine tuberculeuse en raison de l'absence d'une collection des parties molles. La présence d'une destruction fragmentaire vertébrale de l'atlas associée à une tuméfaction des parties molles ne permettait pas d’écarter une pathologie tumorale primitive ou secondaire. Devant ces difficultés diagnostiques, le recours à une biopsie chirurgicale s'avérait indispensable. La certitude diagnostique était alors acquise par l′examen histologique réalisé par biopsie chirurgicale. Les examens anatomo-pathologiques et l'analyse bactériologique confrontés au contexte clinique et radiologique permettent donc la confirmation de tuberculose sous-occipitale dans 99% des cas [5]. La prise en charge conservatrice reste une attitude logique et suffisante pour obtenir la guérison [9, 10]. Le traitement est alors basé sur la chimiothérapie pendant douze mois; complétée éventuellement par une réduction des luxations et une stabilisation orthopédique ou chirurgicale si nécessaire de la charnière cervico-occipitale [6]. La guérison se traduit radiologiquement par une reconstruction osseuse avec fusion des éléments osseux atteints [2]. Le pronostic de cette atteinte est généralement favorable si un traitement adapté est instauré précocement.

## Conclusion

Le mal de Pott du rachis cervical supérieur est exceptionnel. Il expose à de graves complications neurologiques. L'imagerie moderne par le scanner et l'IRM est d'un grand apport diagnostique et permet la surveillance des formes traitées. Le diagnostic doit être précoce, il est confirmé sur des arguments histo-bactériologiques mais parfois retenu sur des éléments de présomption. Le traitement est basé sur l'antibiothérapie antituberculeuse et l'immobilisation du rachis cervical. L’évolution sous traitement est habituellement favorable.
